# Cannabis use in Europe

**DOI:** 10.1007/s00406-025-01972-7

**Published:** 2025-02-14

**Authors:** Eva Hoch, Wayne Hall, Daniel Feingold

**Affiliations:** 1 Department of Psychiatry and Psychotherapy, LMU University Hospital, LMU Munich, D - 80336 Munich, Germany; 2 IFT Institut für Therapieforschung, Centre for Mental Health and Addiction Research, Munich, Germany; 3 Department of Psychology, Charlotte Fresenius University, Munich, Germany; 4 Faculty of Health and Behavioural Sciences, National Centre for Youth Substance Use Research, The University of Queensland, Brisbane, QLD Australia; 5 Queensland Alliance for Environmental Health Sciences, Faculty of Health and Behavioural Sciences, The University of Queensland, Brisbane, QLD Australia; 6 Psychology Department, Achva Academic College, Arugot, Israel

In recent years, the policy debate on how to regulate cannabis use has intensified in Europe. Some countries, e.g. Germany, Luxembourg, Malta, Netherlands, and Switzerland, have adopted a more liberal stance by legalizing medical use and, in some cases, recreational adult cannabis use. It is well established that regular cannabis use, especially that beginning at adolescence, can increase the risks of developing cannabis use disorders [1, 2] and mental illnesses such as psychotic disorders [3]. The effects of regular cannabis use on cognitive development in adolescents and young adults are also of concern [4]. On the other hand, cannabinoids have medical benefits in relieving chronic pain, treating muscle spasticity in multiple sclerosis and serving as an antiemetic in chemotherapy. In addition, the cannabinoid Cannabidiol (CBD) may reduce the frequency of epileptic seizures.

The challenge for European policy makers is in finding ways to obtain the potential benefits of medical uses of cannabinoids without increasing the risks of adverse effects of nonmedical cannabis use. This requires evidence to inform education, research and regulation. It is important that legislation to regulate cannabis is based on scientific evidence about its harms and on the effects of different policies that aim to protect public health while respecting the freedom of adults. Education programs that explain the risks are as important as research that looks at the long-term effects of consumption.

This special issue is a compilation of 13 papers, which address pressing questions in the field of cannabis research and policy with the aim of stimulating discussion on the regulation of cannabis use in Europe and beyond. The papers include the use of cannabis for recreational and medicinal purposes, analysis of cannabis use patterns, health risks, medical benefits, harm-reduction strategies and the treatment of cannabis use disorders in Europe. Six of them are highlighted in this editorial.

Hoch and colleagues [5] explore the evidence on the negative health effects of recreational cannabis use and the safety and effectiveness of medicinal cannabis use. They indicate that regular cannabis use of high THC products can increase mental, gastrointestinal, and cardiovascular problems, increase motor vehicle accidents, and produce cannabis use disorder (CUD). Among adolescents and young adults, chronic cannabis use has been associated with disrupted learning, impaired cognitive performance, reduced educational attainment and an increased risk of CUD, psychosis/schizophrenia, mood and anxiety disorders and suicidal behavior. Clinical trials indicate that the medical use of medicines extracted from herbal cannabis and synthetized cannabinoids can produce small to modest benefits in the treatment of various physical conditions.

Senator and colleagues [6] report an evidence synthesis of medical cannabis research. They analysed 23 systematic reviews on the effectiveness and safety of medical cannabis to discern the extent to which this body of work captured the differing outcomes of medical cannabis products. They found that only a minority of systematic reviews explicitly aimed to describe differences in treatment outcomes for specific medical cannabis products and very few in fact did so. They concluded systematic reviews will need to standardise descriptions of treatments to better capture granular information on medical cannabis treatment outcomes from empirical studies.

Hall and colleagues [7] describe data on the prevalence of cannabis use in Europe and the more limited data on the prevalence of CUD. They summarize evidence on adverse effects of acute and chronic cannabis use and discusses potential health system responses that may reduce these harms. These responses include, among other, public education about the risks of cannabis use; screening and brief interventions for symptoms of cannabis use disorders in primary care medical settings; and specialist treatment for cannabis use disorders that fail to respond to self-help. They also highlight the need for services to address the high rates of comorbidity between CUD, other common drug use disorders, such as alcohol, and common mental disorders, such as anxiety and depression.

Hoch and colleagues [8] updated an overview of cannabis-specific treatments that are available in Europe. They collected data on treatment programs in 27 EU member states, United Kingdom, Norway and Turkey using a mixed-methods approach. They combined data from a quantitative survey of the members of the European National Focal Points of the European Drugs Agency (EUDA) with a qualitative analysis of “Drug Workbooks 2021” and ‘Treatment Workbooks 2020 and 2021’ published by the National Focal Points of the EUDA. They found that half of the 30 countries that provided data reported the provision of cannabis-specific programs. In 13 countries these included face-to-face interventions at regional or local level. In some countries, online-treatment was made available from the beginning of the COVID-pandemic. Automated and brief web-based interventions have emerged to address the needs of many clients in rural areas. More specific forms of treatment for vulnerable target groups (e.g. adolescents, people with mental disorders) are still lacking. Access to cannabis-specific treatment has increased in the past decade in the EU but nationwide availability is still severely limited and there is a lack of specific programs for vulnerable groups.

Connor and colleagues [9] synthesize evidence on the effectiveness of behavioural and pharmacological approaches to treating cannabis use disorders. They integrated findings from high level evidence studies while prioritizing data from a relatively small number of behavioural and pharmacological studies that have been conducted in Europe. They found that Cognitive Behavioural Therapy (CBT) and/or Motivational Enhancement Therapy (MET) produced short-term reductions in the frequency of cannabis use and dependency severity but abstinence was an uncommon outcome. These improvements were typically not maintained nine months after treatment. CBT and MET (or combined CBT + MET) treatments that extended beyond four sessions were more effective than fewer sessions given over a shorter duration. Combining CBT or MET (or combined CBT + MET) with adjunctive Contingency Management (CM) improved therapeutic outcomes. No pharmacotherapies have been approved for the management of cannabis use, cannabis use disorders or cannabis withdrawal but some pharmacological agents have been used ‘off-label’ to manage cannabis withdrawal symptoms.

Feingold and colleagues [10] report that CUD is currently the most common reason for first-time drug-related treatment admission in the European Union. The demand for such treatment has increased in the past decade in almost all European countries. There is a lack of knowledge about factors associated with effective therapy and mechanisms of change among individuals treated for CUDs. The authors use a scoping methodology to review factors that have been shown to contribute to positive outcomes in CUD treatment. These were categorized as either ‘mediators’, (i.e., treatment-related factors associated with the processes or mechanisms through which patients benefit from therapy) or ‘moderators’ (i.e. patient-related characteristics that predicted treatment outcomes). Specific mediators included treatment duration, addressing motivation to change, acquiring coping skills, enhancing self-efficacy, and integrating several therapeutic components. Common mediators included establishing a therapeutic alliance, empathy, clear expectations and cultural adaptation. Moderators in CUD treatment included sex, ethnicity, age-related factors and the presence of comorbid disorders.

Furthermore, in this special issue, cross-country differences and trends in cannabis treatment demand in 30 European countries, cannabis-related treatment demand on the eve of cannabis legalization in Germany, harm reduction strategies for cannabis-related problems, and predictors of an increase in cannabis consumption in Germany are outlined. The course of CUD in adolescents and adults, the link between cannabis and mental disorders, and the concentrations of THC and CBD in different types of cannabis resin are discussed in detail. This provides an actual update on the current field of cannabis research in Europe and beyond.
